# Echocardiographic Assessment of Left Ventricular Function in Type 1 Gaucher's Disease

**DOI:** 10.1155/2010/820843

**Published:** 2010-07-20

**Authors:** Mirta Koželj, Samo Zver, Vesna Zadnik

**Affiliations:** ^1^Department of Cardiology, University Medical Centre Ljubljana, Zaloška 7, 1525 Ljubljana, Slovenia; ^2^Department of Haematology, University Medical Centre Ljubljana, Zaloška 7, 1525 Ljubljana, Slovenia; ^3^Institute of Oncology, Zaloška 2, 1525 Ljubljana, Slovenia

## Abstract

There is predominate opinion among physicians managing type 1 Gauchers' disease (GD) that cardiac involvement is not an issue in these patients. In order to follow this hypothesis, we prospectively investigated 15 adult imiglucerase-treated type 1 GD patients by echocardiography, Doppler, and tissue Doppler echocardiography. This was a case-controlled study with 18 matched healthy volunteers. The obtained data was correlated with the levels of NT-proBNP (brain natriuretic peptide). None of the GD patients had clinical signs of heart disease. In 3 of the 15 patients, we observed echocardiographic signs of aortic and mitral valve calcification. The left ventricular systolic function was within normal limits. Compared to the control group, there was no statistically significant difference observed in the most sensitive indices of left ventricular diastolic function, parameter *E*_*m*_ (*P* = .095), and *E*/*E*_*m*_ ratio (*P* = .097), as demonstrated by tissue Doppler echocardiography. However, there was a positive correlation between the *E*/*E*_*m*_ ratio and NT-proBNP plasma levels (*P* = .009). In conclusion, the prospective echocardiographic study of type 1 GD patients did not validate any left ventricular dysfunction. But, the *E/* *E*_*m*_ ratio showed a strong statistical correlation with the most sensitive indicators of heart failure, NT-proBNP. Research on larger groups of patients and the usage of even more sensitive methods as strain-rate imaging will be necessary to confirm eventual myocardial involvement in GD patients.

## 1. Introduction

Gaucher's disease (GD) is an autosomal recessive inherited defect of lysosomal enzyme beta-glucosidase, which leads to glucocerebroside accumulation in various organs, especially those of the reticuloendothelial system (RES). The fact is that the cells of RES are present in every human organ and consequentially we could expect that virtually every human organ may be involved. Nevertheless, the heart is not thought to be involved in type 1 GD. Meanwhile, there is an exception regarding type 3 GD (characterised by nonacute neurological involvement) in which aortic cusps and mitral leaflets may show calcifications on light microscopy, and Gaucher's cells were confirmed in the lesions by electron microscopy [1–6]. Homozygosity for the D409H mutation has been associated with specific cardiovascular symptoms [6]. Constrictive pericarditis has been described in patients with type 1 GD, but no Gaucher's cells have been found in the pericardium [7–9]. 

There are few reports of pulmonary hypertension in type 1 GD [9]. The first one includes the influential description of pericarditis in Gaucher's disease and pulmonary hypertension [10]. In most cases the alveolar spaces and interstitial tissue were infiltrated with Gaucher's cells. In addition, Gaucher cells may occlude pulmonary capillaries resulting in pulmonary hypertension. Pulmonary disease is a well-known complication of type 1 GD, although its incidence is not well established and its severity varies [11–13].

Left ventricular dysfunction has been described in rare cases of GD [14]. However, systematic echocardiographic evaluation is lacking. We investigated patients with type 1 GD by echocardiography, Doppler, and tissue Doppler echocardiography. We paid special attention to heart valve diseases, eventual signs of pulmonary hypertension, and left ventricular function. Afterwards we correlated the parameters of left ventricular function with the levels of NT-proBNP, the most sensitive biochemical marker of heart failure [15]. 

## 2. Methods

### 2.1. Patients

In our study we included all patients with type 1 GD gathered in the adult's database at University Medical Centre, Ljubljana, which is the only centre for treating adults with this metabolic storage disorder in Slovenia. Glucocerebrosidase gene mutation analysis was performed in all patients, and neither homozygote nor heterozygote was found with present D409H mutation. All type 1 GD patients understudy are receiving imiglucerase for at least five years, and the majority of patients started with imiglucerase seven years before our study was performed. In all of them the therapy is effective, regarding both clinical and laboratory aspects. Before treatment with imiglucerase was instituted, each separate patient was evaluated individually. Nevertheless, every one of them at that time point had at least anemia and thrombocytopenia, hepatomegaly, splenomegaly (if spleen was not already surgically removed), and clear clinical symptoms and radiological signs of bone disease. Their pretreatment chitotriosidase levels were between 2900 to 13300 nmol/L/h. All patients were completely fit for the indications for imiglucerase therapy because of the extent of the disease [16]. At the same time none of the treated patients have had clinical signs of lung involvement characterised by dyspnoea as a leading symptom.

We prospectively studied 15 adults, 7 males and 8 females, aged from 21 to 71 years, with a mean age of 40 (plus or minus 15 years). As the control group, we assembled 18 healthy volunteers matched for sex and age, without any history of cardiovascular disease, diabetes mellitus, or hypertension. Highly trained athletes were excluded. Their physical examinations and resting 12-lead electrocardiograms, were all within normal limits. The study was approved by the Slovenian Medical Ethics Committee and conforms to the principles outlined in the Declaration of Helsinki.

### 2.2. Clinical Investigation

The patients and control subjects underwent detailed clinical evaluation, including a review of medical history and physical examination. Historical data included the position of the patient within the functional classification of the New York Heart Association (NYHA). We also analysed standard 12-lead electrocardiograms. 

### 2.3. Echocardiography

Transthoracic cross-sectional echocardiography, echo-Doppler studies, and tissue Doppler echocardiography were carried out using a Hewlett-Packard Sonos 5500 echocardiographic machine. Dimensions of the left ventricle were measured in the parasternal short-axis view and left ventricular ejection fraction was calculated by Teicholz formula [17, 18]. The degree of tricuspid regurgitation, assessed by colour flow Doppler, was estimated as mild, moderate, or severe by the width and length of the jet [19, 20]. The following diastolic parameters were measured: *E*/*A* ratio, pulmonary vein *S* *D*, *A* velocities, duration of A seen on the pattern of transmitral flow pattern (*A*  dur) and on the pulmonary venous flow (*a* dur). The ratio of duration of *A* to *a* was calculated [21]. 

Pulsed-tissue Doppler echocardiographic images were recorded from the apical 4-chamber view. Maximal systolic (*S*_*m*_), early (*E*_*m*_), and late (*A*_*m*_) diastolic peak velocities were measured. For assessing the velocity of longitudinal excursion of the left ventricle, a sample volume was placed at the mitral annulus at the basal segment of the muscular ventricular septum (*S*_*m*_, *E*_*m*_, *A*_*m*_). For each subject, at least three consecutive cardiac cycles were analysed and averaged. 

### 2.4. Plasma NT-proBNP

Samples were obtained from the antecubital vein in all subjects. Blood was collected into chilled tubes containing preservative, promptly centrifuged, and stored at −70°C until final analysis. Levels of NT-proBNP in the serum were measured by the electrochemiluminescence immunoassay using a Roche Elecsys immunoassay analyzer (Roche Diagnostics GmbH D-68298 Mannheim). The measuring range of 5 to 35.000 ng/L was defined by the lower detection limit and the maximum of the master curve. The cutoff value for normality was 125 ng/L [22]. 

### 2.5. Statistical Analysis

Numerical variables are presented as the mean ± SD. The parameter values were tested for normal distribution with the Kolmogorov-Smirnov test. Associations among normally distributed variables were evaluated by calculating Pearson correlation coefficients, and the Spearman correlation coefficient was used when differences were not distributed normally. Statistical analyses were performed with the SPSS software package (version 13.0). 

## 3. Results

### 3.1. Clinical Investigation and ECG

All enrolled patients had normal baseline cardiovascular status. None of the patients developed overt heart failure, and all of them were in functional class I by NYHA. All patients were in regular sinus rhythm without any signs of right ventricular hypertrophy. 

### 3.2. Echocardiography

In one GD patient, there were echocardiographic signs of minimal aortic valve calcification, another one had minor calcifications of the mitral annulus while one patient showed both aortic cusps and mitral annular calcifications. All three patients were the oldest patients in the group (56, 63, and 71 years). Some mild calcifications of aortic cusps were found only in the oldest control subject (71 years of age). 

No echocardiographic abnormalities have been seen in the pericardial area. None of the patients showed any signs of pulmonary hypertension. Only 2/15 (64 and 21 years) patients had no more than noticeable tricuspid regurgitation with tricuspid regurgitation flow velocity less than 2.5 m/s. Echocardiographic parameters of left ventricular systolic and diastolic functions are indicated in Table 1.

### 3.3. Correlation between Cardiac Markers and Echocardiographic Variables

Correlation was assessed between biochemical (NT-proBNP) and echocardiographic parameters of left ventricular diastolic dysfunction (*E*/*A* and *E*/*E*_*m*_). There was a significant positive correlation between NT-proBNP levels and the ratio *E*/*E*_*m*_(*P* = .009). The correlation between NT-proBNP levels and *E*/*A* ratio revealed borderline statistical significance (*P* = .091).

## 4. Discussion

In the control group of 15 patients diagnosed with type 1 GD, there was no evidence of significant cardiovascular manifestations of disease. This is in accordance with current information from the literature, with the exception of some clinical case reports [1–6]. The latter usually refer to mutation D409H or the neurological forms of GD. None of our patients had accompanying cardiovascular diseases, and all had normal physical capability for their age and normal sinus rhythm on electrocardiogram. Taking into consideration clinical signs and echocardiograhic examinations, none of the control group had pericardial changes such as pericardial effusion or constrictive pericarditis. Regarding the data from the literature, the pericardial involvement could be due to the organization of hemorrhagic pericardial effusion, yet patients with Gaucher's disease have hemorrhagic tendency [7, 8, 23–25]. 

During careful examination of the valves, we observed echocardiographic signs of aortic and/or mitral valve calcification in three patients. Considering the degrees of calcification and age of the patients, we can conclude that they were due to degenerative valvular changes, but this cannot be confirmed with certainty based only on echocardiograms. In our patients, there were aortic valve calcifications on the basal region of the cusps and on the mitral valve ring, which is quite characteristic for atherosclerotic valvular changes. In all of our patients, the valves were functionally normal. We observed mild aortic valve calcification only in the oldest patient in the control group. On the basis of only echocardiographic evidence, it is difficult to conclude the aetiology of the valvular changes. 

None of our patients had signs of pulmonary arterial hypertension or signs of cor pulmonale based on echocardiographic assessment. This is important because, as possible trigger for pulmonary artery hypertension in type 1 GD, even enzyme replacement therapy was mentioned. Namely, there are two reports from the literature describing four patients with new onset pulmonary artery hypertension after therapy with human placental tissue-derived alglucerase began [26, 27]. We have found no such data in the literature regarding therapy with imiglucerase and, as expected, clinical/echocardiographic data obtained from our patients proved imiglucerase very likely to be safe. This can be interpreted with clear structural and manufacturing differences between these two drugs. For instance, regarding alglucerase, no procedure has been shown to be totally effective in removing viral infectivity, and pulmonary artery hypertension could be a consequence of unrecognised viral infection. 

We can conclude that none of our patients had pulmonary hypertension which complies with the clinical picture of our patients. None of the treated patients had clinical signs of pulmonary manifestations of GD (symptomatic lung involvement). Also, none had signs of right ventricular hypertrophy on electrocardiogram. The NT-proBNP concentrations in our patients were within normal limits. Elstein et al. proved that NT-proBNP concentrations in Gaucher disease correlate with the severity of pulmonary hypertension assessed by the Doppler echocardiography (tricuspid gradient) [28]. And as it is known that a group of 15 patients are probably too small to compare the incidence of changes to the pericardium, valves, and pulmonary arterial hypertension with other authors. In fact, according to available sources in the literature, such a prospective echocardiographic study has not yet been performed. Finally, the low incidence of Gaucher's disease poses a serious obstacle. 

Our aim was to accurately determine left ventricular diastolic and systolic function in patients with type 1 GD, which until now has not yet been systematically researched. Systolic function was evaluated using conventional echocardiography by calculating the left ventricular ejection fraction and with tissue Doppler echocardiography. Both methods have shown normal systolic function that does not differ from the control group of healthy subjects. Considering the basic disturbance that could affect the myocardium, since this is an infiltrative disease (similar to pattern of involvement that can be seen in the lungs), one would expect left ventricular diastolic dysfunction [29]. Doppler parameters showed a tendency towards diastolic dysfunction, but the differences between the GD patients and control group were entirely atypical. Even the most sensitive parameters of left ventricular diastolic function, *E*_*m*_ and *E*/*E*_*m*_ ratio, using tissue Doppler did not significantly confirm diastolic dysfunction of the left ventricle in the group of GD patients. We noticed a slight and statistically not significant increase in the *E*/*E*_*m*_ ratio in GD group although it stayed in normal limits. In general, significantly increased *E*/*E*_*m*_ ratio could be a sign of increased pressure in the left chamber which is a reflection of left ventricular diastolic dysfunction [30–33]. 

The most interesting fact is that there is a significant positive correlation between the *E*/*E*_*m*_ ratio and the NT-proBNP concentration, which is the most sensitive marker for neurohumoral activation of heart failure. There was also a nonstatistically significant correlation between the *E*/*A* ratio (a parameter for left ventricular relaxation disturbance) and the NT-proBNP concentration. The NT-proBNP concentration was within normal limits in both groups. 

In the prospective echocardiographic study of type 1 GD patients, we did not find signs of constrictive pericarditis, pulmonary arterial hypertension, and also there were non conclusive GD characteristic heart valve changes, which are most frequently described in GD. We observed some tissue Doppler parameters that could indicate that GD patients have left ventricular diastolic dysfunction but the differences between GD group and controls did not reach statistical significance. In particular, the *E*/*E*_*m*_ ratio showed a strong statistical correlation with the most sensitive indicators of heart failure, NT-proBNP. Based on our results, we cannot conclude that GD patients develop left ventricular diastolic dysfunction that can be detected with echocardiography. Research on larger groups of patients and the usage of even more sensitive methods as strain rate imaging will be necessary to confirm eventual myocardial involvement in GD patients. 

## Figures and Tables

**Table 1:** Echocardiographic parameters in patients and controls.

	Patients *n* = 15	Control subjects *n* = 18	*P* value
LV EF (%)	70.7±7.51	64.2 ± 3.42	NS
*E* (cm/s)	88±17	91 ± 19	NS
*A* (cm/s)	66 ± 13	65 ± 16	NS
*E*/*A*	1.36 ± 0.35	1.46 ± 0.44	NS
*S* (m/s)	0.59 ± 0.09	0.59 ± 0.14	NS
*D* (m/s)	0.56 ± 0.11	0.63 ± 0.14	NS
*S*/*D*	1.07 ± 0.27	0.96 ± 0.25	NS
*A* dur (ms)	125 ± 19	139 ± 26	NS
*a* dur (ms)	96 ± 19	101 ± 22	NS
*A*/*a*	1.34 ± 0.27	1.44 ± 0.32	NS
*S*_*m*_ (cm/s)	8.2 ± 1.2	8.5 ± 0.9	NS
*E*_*m*_ (cm/s)	11.2 ± 4.1	13.3 ± 2.8	0.095*
*A*_*m*_ (cm/s)	9.8 ± 2.0	9.4 ± 1.9	NS
*E*/*E*_*m*_	8.8 ± 3.3	7.3 ± 1.8	0.097*
NT-proBNP (ng/L)	72 ± 63	50 ± 34	NS

Echocardiographic parameters: LV EF = left ventricular ejection fraction *E* = flow velocity during early diastolic filling, *A* = flow velocity during atrial contraction, late diastolic filling, *E*/*A* = *E* wave/*A* wave ratio, *A* dur = duration of mitral *A*-wave, *a* dur = duration of retrograde a-wave in pulmonary venous flow, *A*/*a* = ratio between the duration of mitral *A*-wave and duration of *a*-wave in pulmonary venous flow, *S*/*D* = ratio between systolic and diastolic pulmonary venous flow, *S*_*m*_ = mitral annulus systolic tissue velocity, *E*_*m*_ = early mitral annulus diastolic tissue velocity, *E*/*E*_*m*_ = ratio in early mitral diastolic flow and early mitral annulus diastolic tissue velocity, NT-proBNP = amino terminal pro brain natriuretic peptide, NS-statistically nonsignificant, and *statistically borderline values.
